# Supplementary material for: Highly adaptable deep-learning platform for automated detection and analysis of vesicle exocytosis
Source: Nat Commun. 2025 Jul 12;16:6450. doi: 10.1038/s41467-025-61579-3 (PMC12255801; doi:10.1038/s41467-025-61579-3)
Supplement: Supplementary file 1 — Supplementary Information [file 41467_2025_61579_MOESM1_ESM.pdf]

Highly adaptable deep-learning platform for automated detection and analysis of vesicle exocytosis

Abed Alrahman Chouaib[1], Hsin-Fang Chang[1], Omnia M. Khamis[1], Santiago Echeverry[2], Lucie Demeersseman[3], Sofia Elizarova[4], James A Daniel[4,8], Salvatore Valitutti[3], Sebastian Barg[2], Constantin Pape[5,6], Ali H. Shaib[7]* and Ute Becherer[1]*

Supplementary Figures

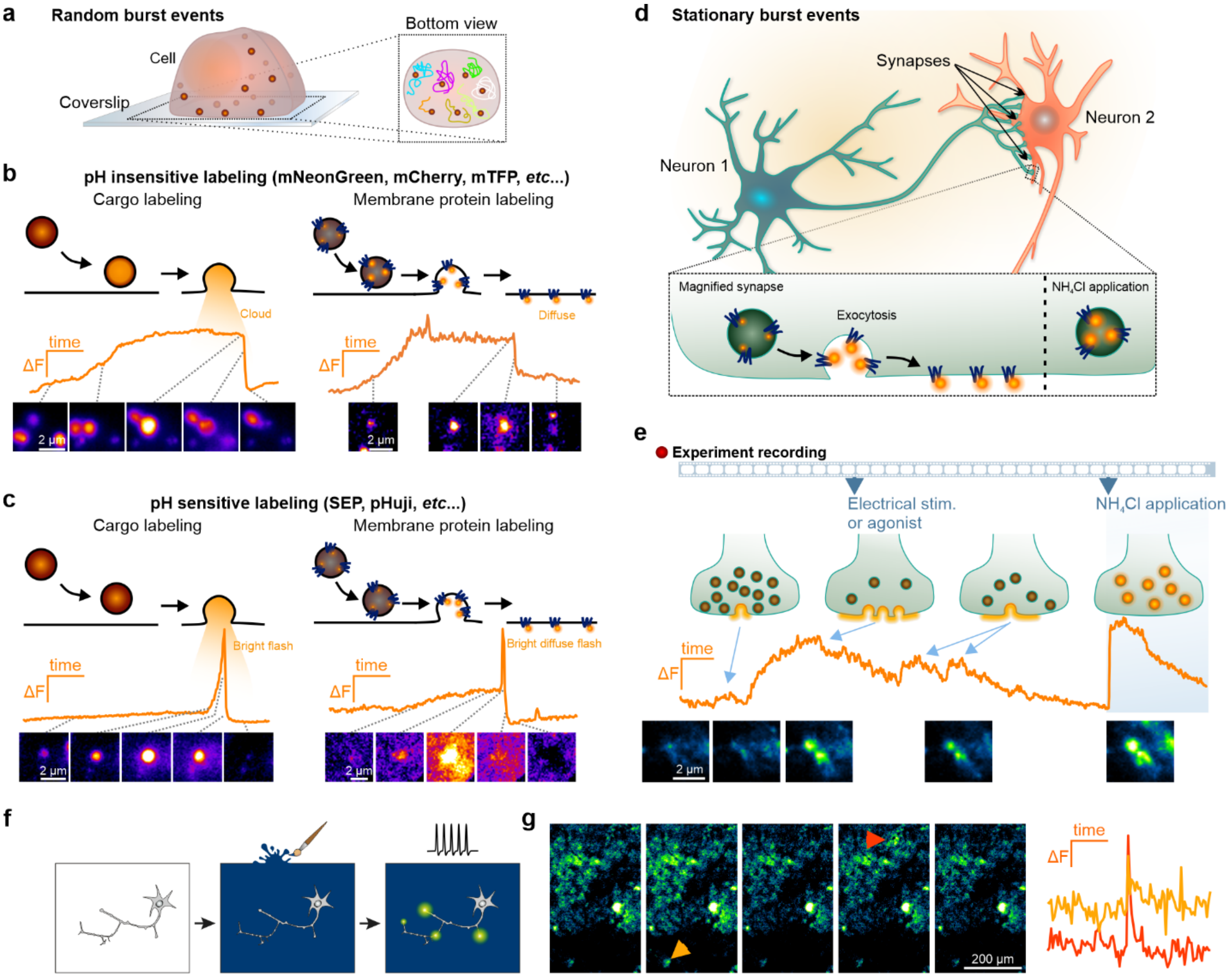

Supplementary Figure 1. Exocytosis measurements and characteristics across different systems. **a**, Schematic representation of a cell in which secretory vesicles/granules, are labeled by a fluorescent marker. They are distributed throughout the cell (left) and move over time (right). **b-c**, Top row: Schematic representation of the successive events occurring during stimulated exocytosis of individual granules. Middle row: Fluorescence intensity over time of a single granule shown below. Bottom row: Snapshots of the granule during exocytosis. The time points of the snapshots are indicated by a stippled line on the graph above. Displayed are examples of the fusion of lytic granules in cytotoxic T cells. **b**, Examples of the exocytosis of granules labelled by pH insensitive fluorescent proteins that are either bound to a cargo

protein (right, granzyme B) or a membrane protein (left, synaptobrevin2). **c**, Examples of the exocytosis of granules labelled by pH sensitive fluorescent proteins such as the super ecliptic pHluorin (SEP) that are either bound to a cargo protein (right, granzyme B) or a membrane protein (left, synaptobrevin2). Note that with the pH sensitive probe, fusion of the granule is accompanied by a sharp increase in fluorescence intensity due to neutralization of the vesicular lumen. **d**, Scheme of synaptic transmission measured with synaptophysin-SEP (SypHy). The pH-sensitive fluorophore SEP is located in the acidic environment of the vesicle, which quenches its fluorescence. Upon exocytosis, SEP is exposed to the neutral pH (7.4) of the extracellular medium lightening it up, while NH_4^+-containing medium neutralizes the lumen of all the vesicles making them visible. **e**, Exemplary measurement of synaptic transmission with SypHy. Top: scenarios that may occur at individual synapses with their specific fluorescence intensity variation over time (middle). The lower row exhibits snapshots of this synapse at the time points of the scenarios shown in the upper row. During non-synchronous exocytosis, small and brief increases in fluorescence are observed when a few vesicles fuse with the plasma membrane. Electrical or agonist stimulation causes simultaneous exocytosis of many vesicles, leading to a sustained rise in fluorescence while SypHy remains in the plasma membrane. The fluorescence intensity decreases upon the endocytosis of SypHy and subsequent reacidification of the vesicle. NH_4^+ induces maximum fluorescence intensity increase. Note that changes in fluorescence intensity occur at fixed small locations, i.e. the synapse. **f**, Illustration of a dopaminergic neuron culture surrounded by a ‘painted’ fluorescent dopamine nanosensor surface (‘AndromeDA’). **g**, Stimulation of the neuron leads to the release of DA, which interacts with AndromeDA. This interaction triggers the nanosensor fluorescence, allowing detection of the spatiotemporal pattern of DA release and diffusion (left), and to analyze release kinetics (right).

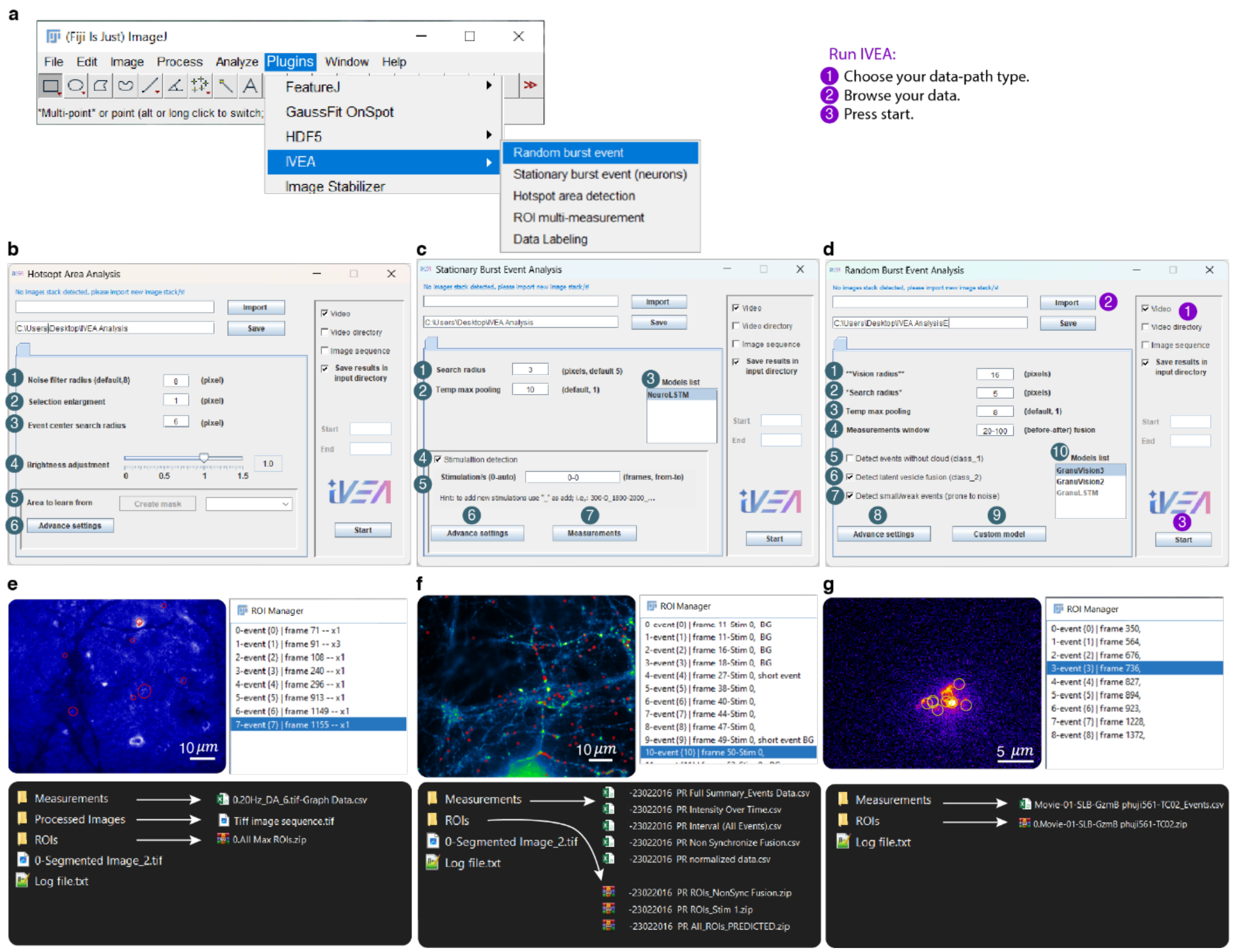

Supplementary Figure 2. IVEA GUI. a, Plugin location in Fiji. **b**, Hotspot Area Events Analysis: Noise filter used for extracting events from the image, the higher it goes the less sensitive detection will be (This filter doesn't affect the measurements). 2- Enlarge the mask by n pixels. 3- Search radius to identify the events identity. 4- Brightness adjustment sensitivity (used for image intensity fluctuation correction). 5- Select an area for the program to learn from. 6- Advanced settings containing control parameters that are mostly determined automatically by the software. **c**, Stationary burst events analysis: (1**c**,2**d**)-Search radius is the size of the ROI radius around an event. (2**c**,3**d**)- Temporal max pooling, reduces the video using moving maximum intensity projection, i.e., 1 means no frame reduction. (3**c**,10**d**)- List of available pretrained models offered by IVEA. 4- Enables stimulus detection if checked, otherwise simulation will be excluded. 5- Simulation timing from a to b (a-b). a or b is determined automatically if set to 0. 6- Advanced settings for stationary burst event default parameters. 7- Measurement settings for output data. **d**, Random burst events Analysis: 1- Vision radius is the radius for the image patch size for the network to visualize 4- Measurement time interval from a to b (a-b). 5- Include pH-insensitive events. 6- Include latent garnule fusion.7- Enabling the "small or weak events" option deactivates the filter that excludes weak fluorescence signal intensity, which is comparable to the intensity of noise events. 8- Advanced settings for random burst event default parameters. 9- Controls the export of training data and the use of custom neural networks. **e**, Hotspot area events analysis output. **f**, Stationary burst events analysis output. **g**, Random burst events analysis output.

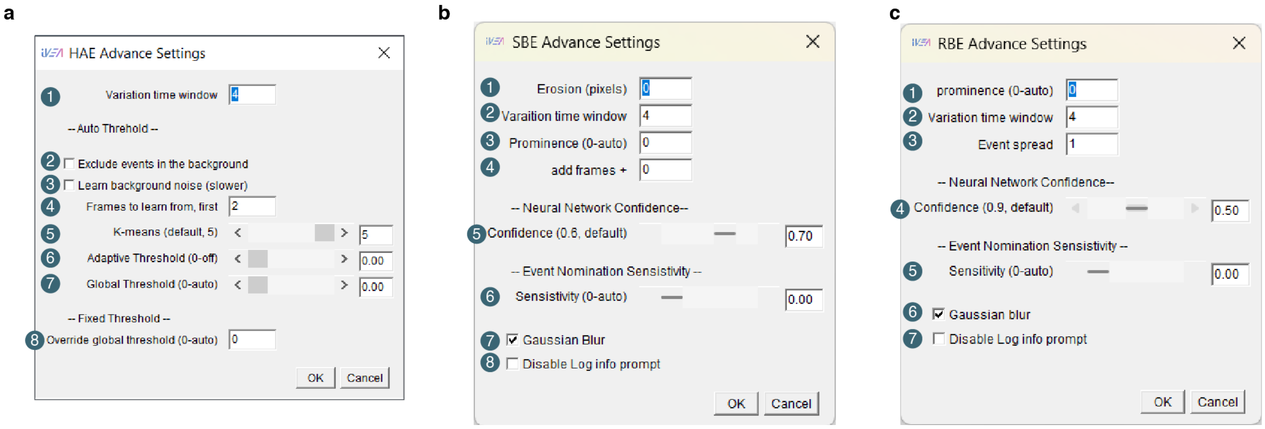

Supplementary Figure 3. IVEA advanced settings GUI. a, Hotspot Area Events advanced settings: time frame variation interval. 2- Exclude events that occur in the background. 3- Analyze noise from the background. 4- Specify the frame used by IVEA to determine the threshold. 5- Number of layers for image segmentation. 6- Adaptive threshold used to locally segment hotspot areas and extract the brightest regions. 7- Threshold sensitivity is automatically detected, 8- Override global threshold. **b**, Stationary burst events advanced settings: **1-** Erosion used only if user want to eliminate small events. (2**b**,2**c**)- Time frame intensity variation interval. (3**b**,1**c**)- **"**Find Maxima" prominence. 4- Add frames to analyze by the neural network. (5**b**,4**c**)- Neural network confidence level, threshold the logit prediction values. (6**b**,5**c**)- Detection sensitivity controls the threshold that filters region of interest at the noise level. (7**b**,6**c**)- Gaussian blur filter the duplicate images at the detection stage. (8**b**,7**c**) Disable the Log information window prompt. **c**, 3- Increases the sigma of the event's spread to suppress the duplicates, default is 0, but for wide event spread it is recommended to be increased by 1.

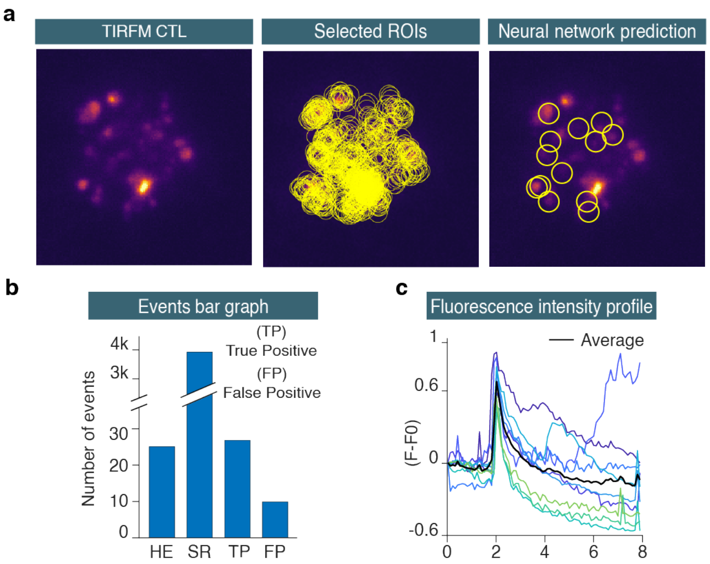

Supplementary Figure 4. CTL analysis – Exocytosis of LysoTracker Red labeled lytic granules. a, The first image shows the raw TIRFM image of a CTL cell in which the lytic granules were labeled with LysoTracker Red. The second image is the overlay of the selected regions of interest prior to import into the neural network. The final image displays the overlay of the neural network's predictions, indicating events identified as true. **b**, The bar graph illustrates the comparison of outcomes between the human expert (HE) and the IVEA software. The selected ROIs are indicated by "SR." The average recall was 100 ± 0%, the precision was 66.59 ± 6.24% and the F1 score was 79.27 ± 4.50% (n_{cell}=4). **c**, The graph shows the intensity profile of the fusion events detected as true positives. Images acquired at the Cancer Research Center of Toulouse, France. Source data are provided as a Source Data file.

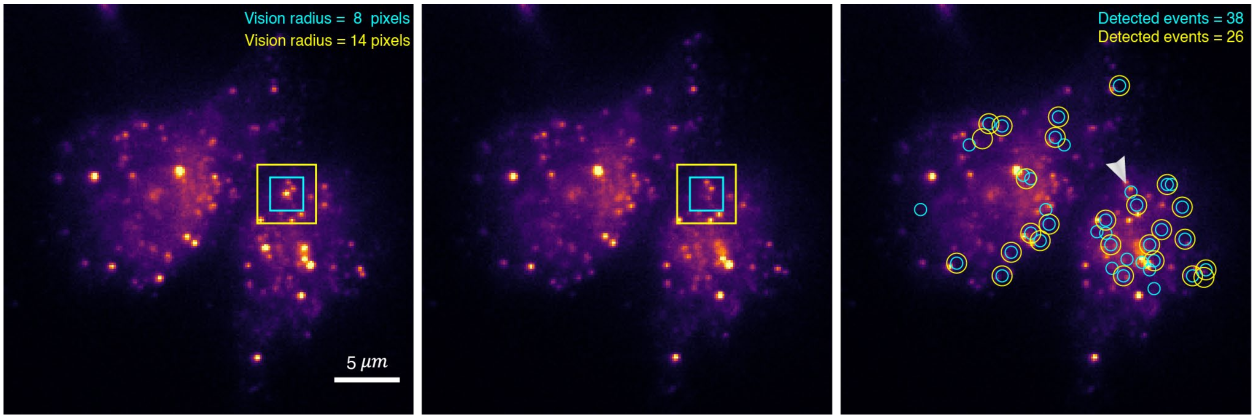

Supplementary Figure 5. Impact of vision radius adjustment on the output results. This figure demonstrates how varying the vision radius of the neural network input image patch size affects exocytosis detection. Two vision radii were tested: 8 pixels (cyan) and 14 pixels (yellow). The first image displays a granule centered within the overlay masks, representing the input patch for classification. The second image shows the disappearance of the granule, indicating an exocytosis event. The third panel presents the detected events for both vision radii, with 38 events identified using the 8-pixel radius (cyan) and 26 events detected with the 14-pixel radius (yellow). These differences highlight how the choice of vision radius impacts the sensitivity of event detection. Pixel size corresponds to 160 nm in physical space.

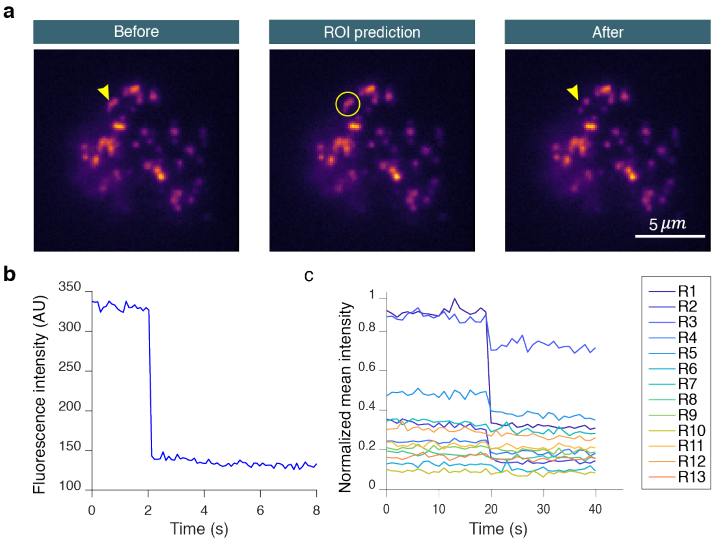

Supplementary Figure 6. Chromaffin cell analysis. a, Images of a chromaffin cell showing an exocytotic event detected by IVEA. Granules are stained through over-expression of NPY-mCherry (pH-insensitive, data from[1]). **b**, Fluorescence intensity profile of the event over 80 frames. **c**, Exocytic pattern is different from another pattern the LSTM network was trained on. These data were better analyzed with the eViT network.

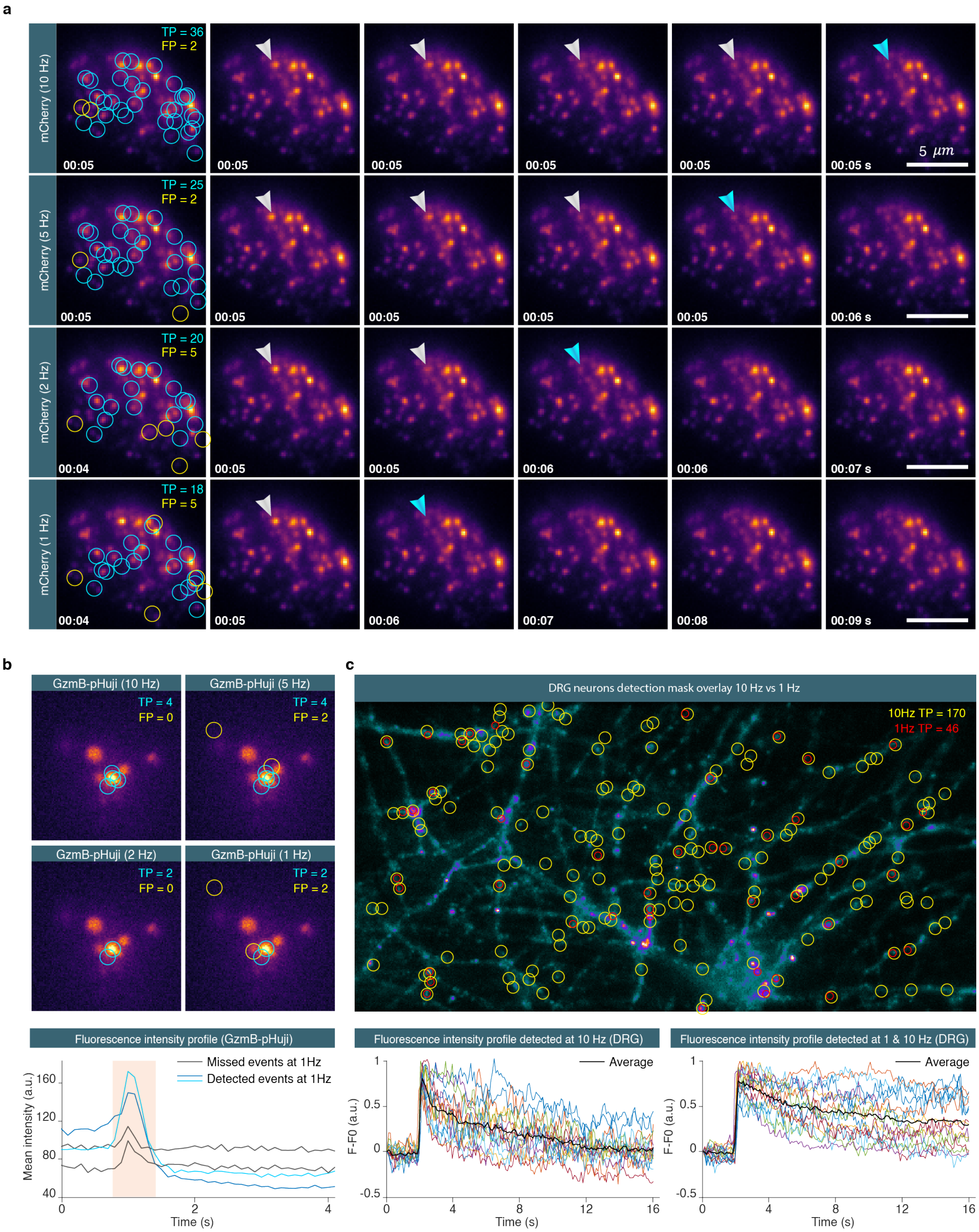

Supplementary Figure 7. Down-sampling Analysis of IVEA Detection Performance.

This figure illustrates the effect of frequency down-sampling on IVEA's ability to detect exocytosis events across different acquisition rates. **a,** Presents a time series of images from a chromaffin cell stained with

NPY-mCherry, showing an exocytosis event occurring at 5 seconds. Each row corresponds to a different imaging frequency: 10 Hz, 5 Hz, 2 Hz, and 1 Hz. The first image in each row displays the raw data overlaid with IVEA-detected regions of interest (ROIs), where cyan circles indicate true positives (TP) and yellow circles represent false positives (FP). Subsequent images in each row show the temporal evolution of the event at the respective frequency. **b**, Displays IVEA's detection results for control (CTL) cells stained with GzmB-pHuji, analyzed at 10 Hz, 5 Hz, 2 Hz, and 1 Hz. The detection overlays follow the same convention, with cyan marking TP events and yellow marking FP events. The accompanying intensity profile graph highlights the fluorescence signal of detected and missed fusion events in the 1 Hz dataset, demonstrating how temporal down-sampling affects event detection. **c,** Presents the comparative detection of exocytosis events in DRG neurons at 10 Hz versus 1 Hz. The overlaid detection masks show the yellow ROIs for TP events detected at 10 Hz, while red ROIs indicate TP events detected at 1 Hz. The two graphs below illustrate fluorescence intensity profiles of detected events: the left graph represents events detected at 10 Hz but missed at 1 Hz, while the right graph shows events detected at both 10 Hz and 1 Hz, highlighting the impact of reduced acquisition frequency on event identification. This analysis confirms that IVEA remains capable of detecting exocytosis at lower frequencies, but as the acquisition rate decreases, fast fusion events are naturally eliminated due to reduced temporal resolution, making them undetectable both manually and computationally.

a

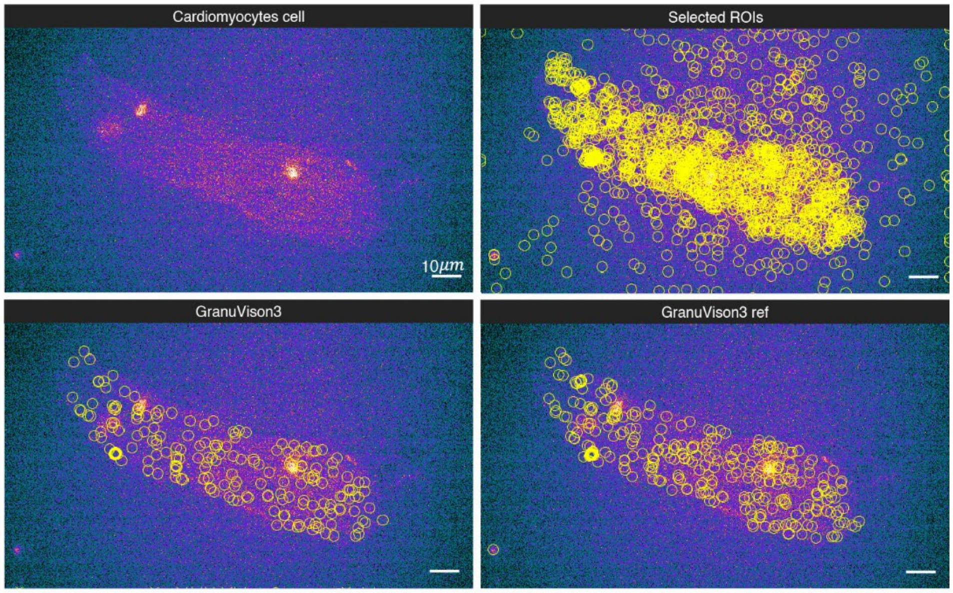

b

IVEA parameters used in this analysis	Model	GranuVision3	GranuVision3 ref
Vision radius: 12<br>Search radius: 4<br>Prominence: 30<br>Event spread: 1<br>Model confidence: 0.5<br>Sensitivity: 0.5	TP	200	298
	FP	1	2
	Selected ROIs	1558	1558

Supplementary Figure 8. GranuVision3 refinement training on calcium sparks results.

a. Shows a mouse cardiomyocyte in which calcium sparks have been measured using Fluo4 imaging. Top left displays an exemplary frame in which the cardiomyocyte exhibit three sparks. Top right presents the selected regions of interest (ROIs) prior to importation into the neural network. Bottom left, shows the raw data overlaid with IVEA-classified ROIs using the original GranuVision3 model. Bottom right displays IVEA-classified ROIs using the refined GranuVision3 model. Pixel size was 0.217 x 0.217 µm and the acquisition speed was 124 frames per second **b.** Table presenting the output of IVEA using GranuVision3 before and after refinement. Note that the refinement largely increased the number of correctly classified sparks (TP) while increasing only by one the number of FP.

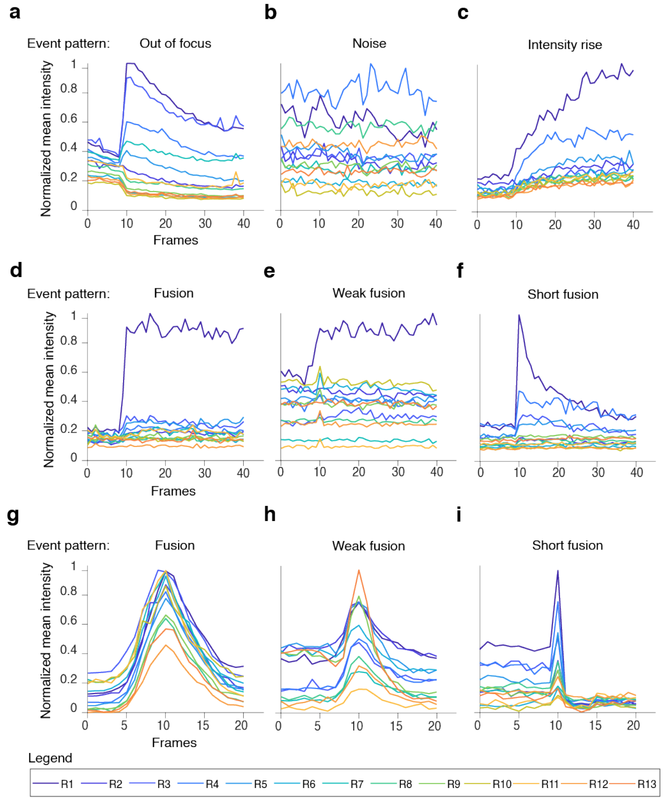

Supplementary Figure 9. Events' patterns for LSTM network. a, Out of focus patterns for DRG neurons. **b**, Random noise. **c**, intensity rise. **d**, DRG neurons fusion event. **e**, DRG neurons weak fusion event. **f**, DRG neurons short fusion event. **g**, T cell fusion event. **h**, T cell weak fusion event pattern. **i**, T cell short fusion event pattern.

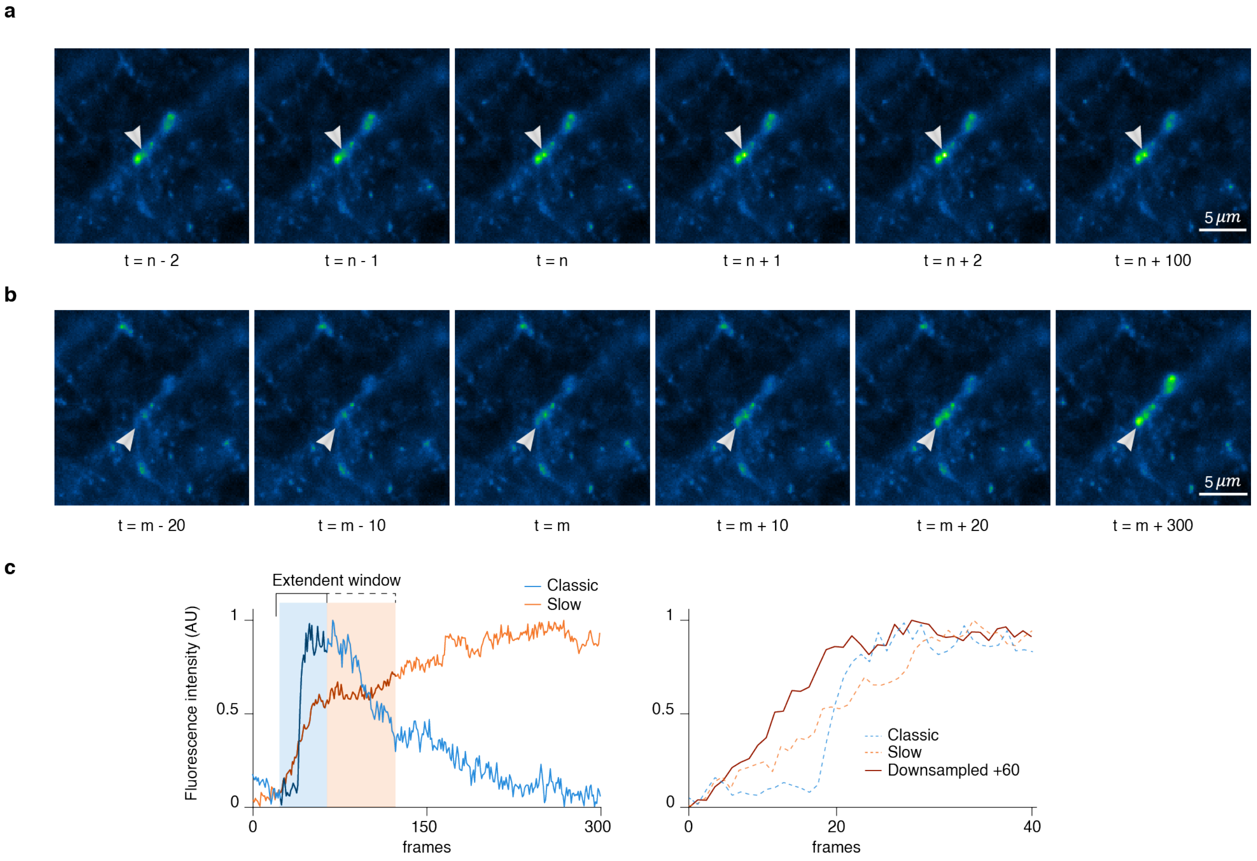

Supplementary Figure 10. Slow vs classic fusion events. a, Represents the timeline of classic fusion event. **b**, represent the timeline of slow fusion event. **c**, The first graph represents the event intensity profiles over 300 frames. The blue shade area is the default measurement window, while the orange shade area is the extended window adjusted by the user. The second graph compares the down-sampled signal with the classic fusion event and the rise intensity event.

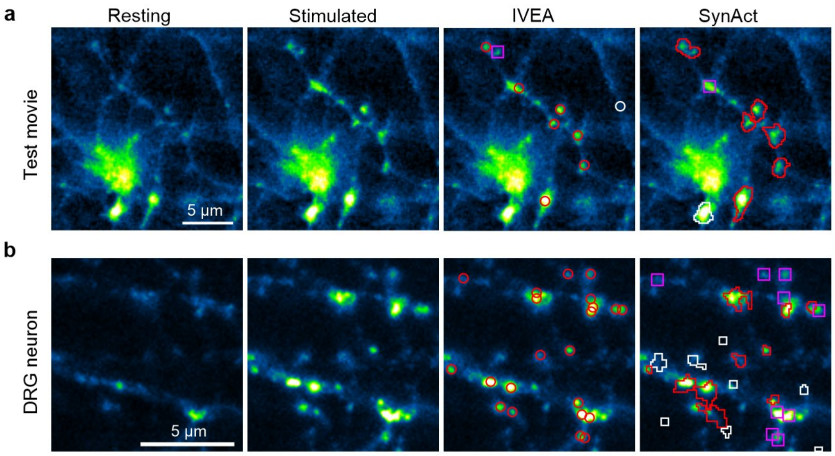

c

SynActJ Settings			Test movie	DRG neuron
Background		Gauss sigma	4	4
		Selected threshold	MinError	MinError
Boutons :	Detect	LoG sigma	0.5	0.5
		Prominence	0.005	0.005
	Segment	Gauss sigma	1.0	0.5
		RollingBall radius	30	20
		Selected threshold	Triangle	IJ -IsoData
		Erode Mask	OFF	ON

Supplementary Figure 11. IVEA is superior to SynActJ in detecting active synapses. a-b, Comparison of IVEA and SynActJ for the detection of active synapses. From left right are shown images of the neurons at rest, during stimulation, with the labelled synapses detected by IVEA and the detected synapses by SynActJ. Shown in red are the overlaid ROIs of the true positive events, in white are the false positive events and the magenta squares are the missed events. **a**, Test movie provided with SynActJ. SynActJ settings were default values as recommended. For IVEA analysis, due to the small sequence size, the first and the last frames of the movie had to be duplicated so that the sequence reached 60 frames in length. **b**, Movie acquired with DRG neurons over-expressing SypHy. For this analysis the movie was restricted to 300 frames encompassing the stimulation at frame 60. The raw data were analyzed by IVEA whereas frame reduction and a Gaussian blur of 0.5 had to be applied to enable SynActJ to detect any event. SynActJ settings were adjusted iteratively to improve detection. **c**, SynActJ settings used to analyze the movies shown in ***a*** and ***b***. Default settings were used for IVEA's analysis for the movie shown in (**b**), while for the movie shown in (**a**), the automatic parameters were set off, the prominence was set to 10 and the sensitivity to 1.

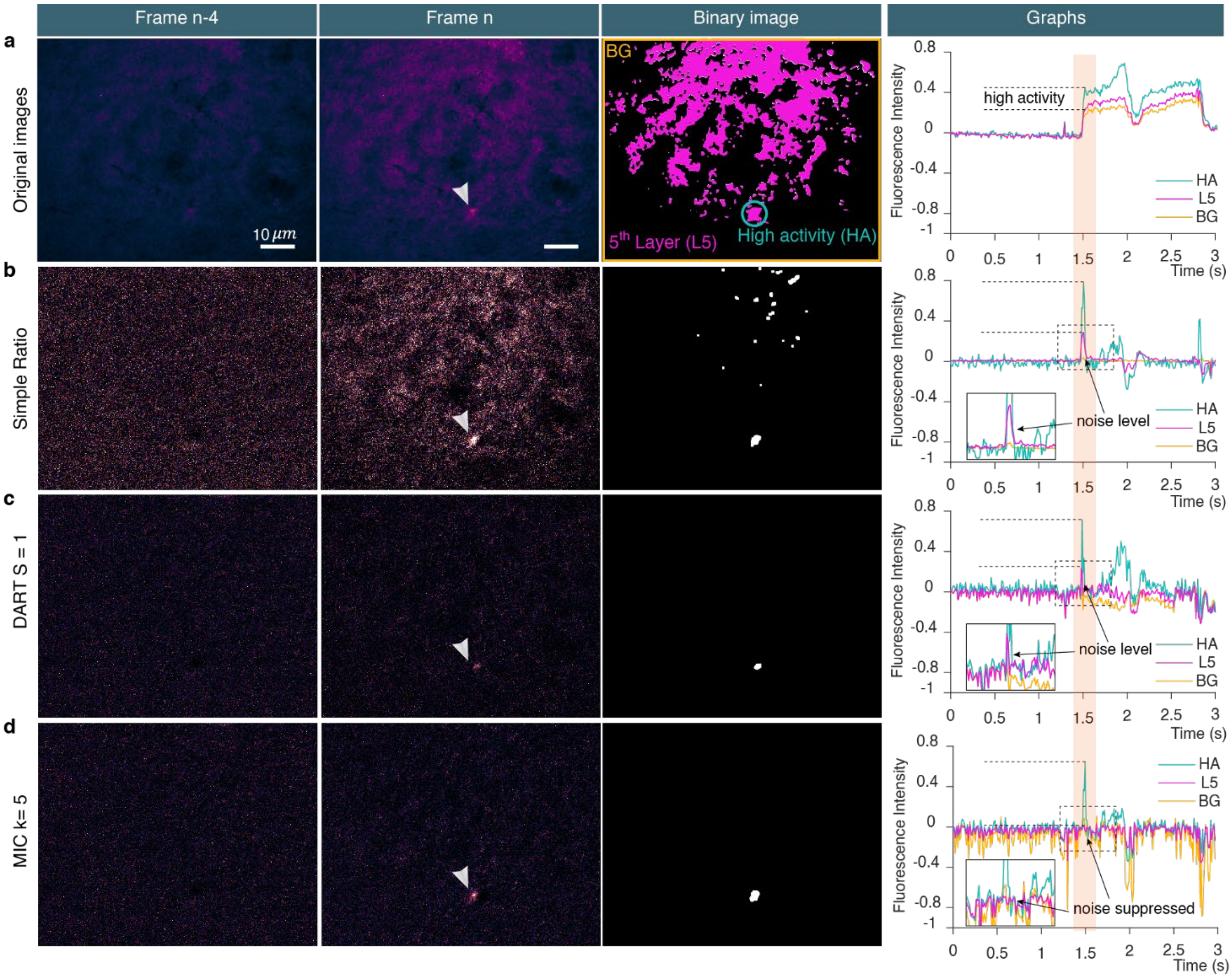

Supplementary Figure 12. Multi-layer intensity correction (MIC). The figure displays the results of three different algorithms, which have been employed to address non-uniform regional fluctuations in fluorescence intensity in videos. The first column represents an image without fluctuations. The middle column corresponds to an image containing a region of high activity and a background with fluorescence intensity fluctuations. The last three images in the third column show the binary images of the second column. They were generated by applying a triangular threshold, followed by morphological reconstruction comprising erosion, a median filter, and then dilation. The last column represents the fluorescence intensity profiles across three distinct regions displayed in the first binary image. These include the fifth segmented layer highest k-means cluster value (L5), represented in pink; the high activity region (HA), in blue; and the background (BG), in orange. The inset is a magnification of the curves delineated by a stippled line. **a**, Shows the raw images, while the binary image represents the L5 of the raw clustered image using k-means (k=5). The graph shows the high activity of the HA region compared to the L5 and BG regions over time. **b**, Impact of using simple ratio on non-uniform fluctuations in the image. The binary image shows how simple ratio failed to eliminate the false regions. The graph illustrates the presence of remaining noise in the measurement of the HA region. **c**, This panel shows the results of the previous method DART. Note that it partially addresses the non-uniform fluctuations. **d**, MIC (k=5) was able to suppress the noise entirely while preserving the HA.

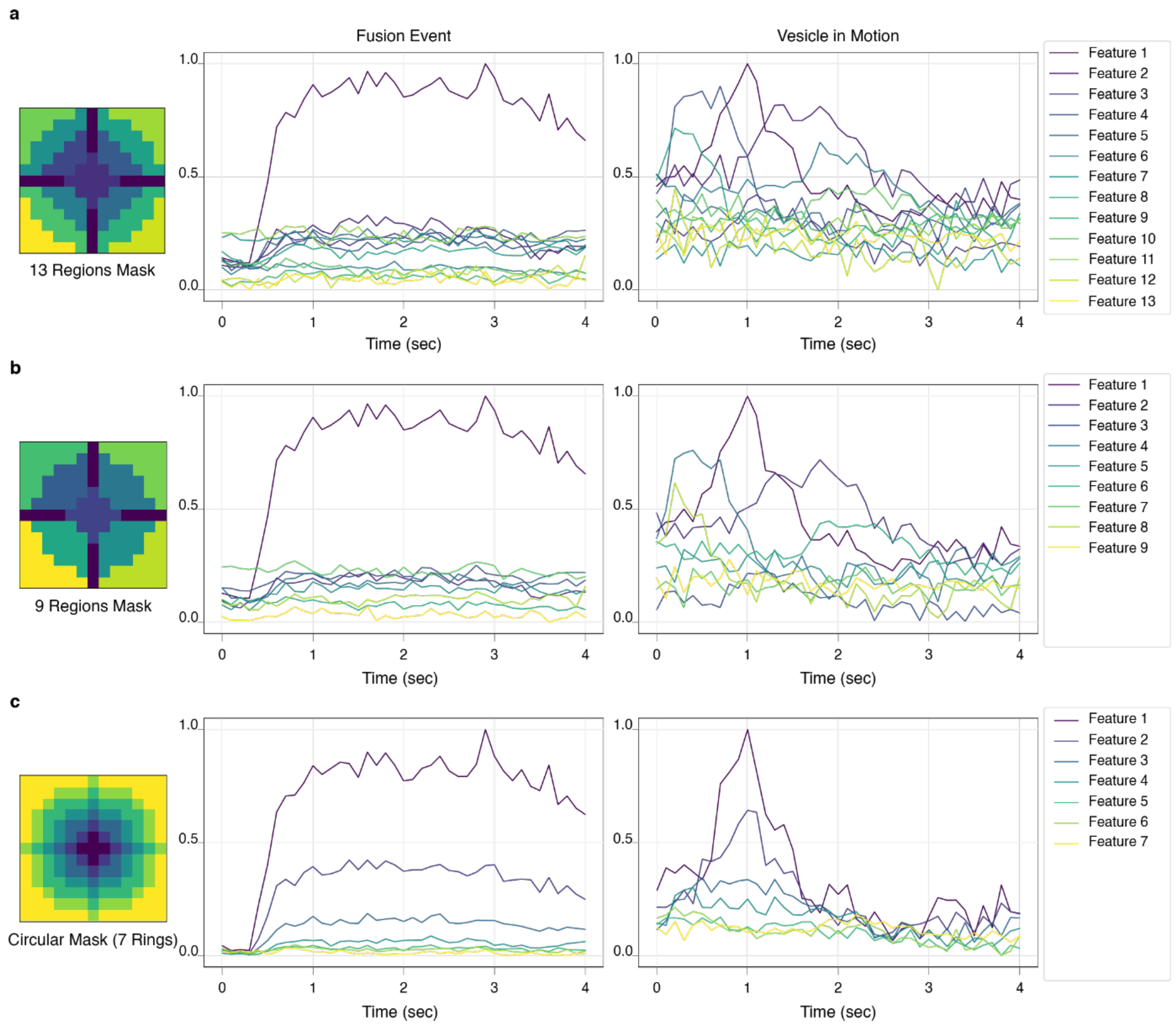

Supplementary Figure 13. Visualization of signal patterns using different mask implementations for the LSTM network. This demonstrates how changing the regions in an area surrounding an event impacts the signal features, the first column displays the mask shape, the second the signal features of fusion event, and the last shows features of a vesicle movement. **a**, Represents the current 13 regions mask applied to the random burst algorithm. **b**, Shows the reduced number of regions for mask of 9 regions. **c**, Represents 7 circular rings around an event instead of symmetrical regions.

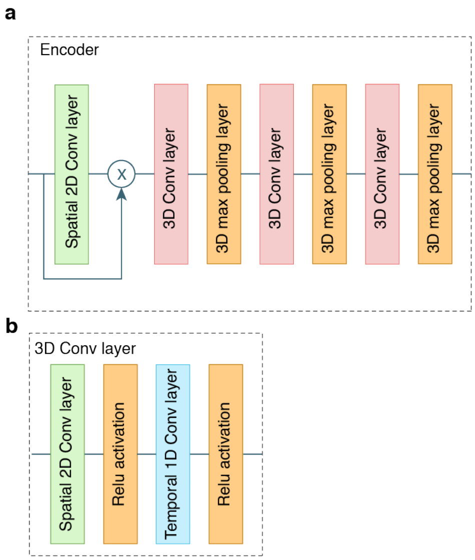

Supplementary Figure 14. Encoder architecture. a, Shared CNN layers, 2D spatial convolution followed by 3D conv layer 3D max pooling. **b**, 3D conv layer consisting of separate 2D spatial convolution followed by 1D temporal convolution followed by ReLU activation.

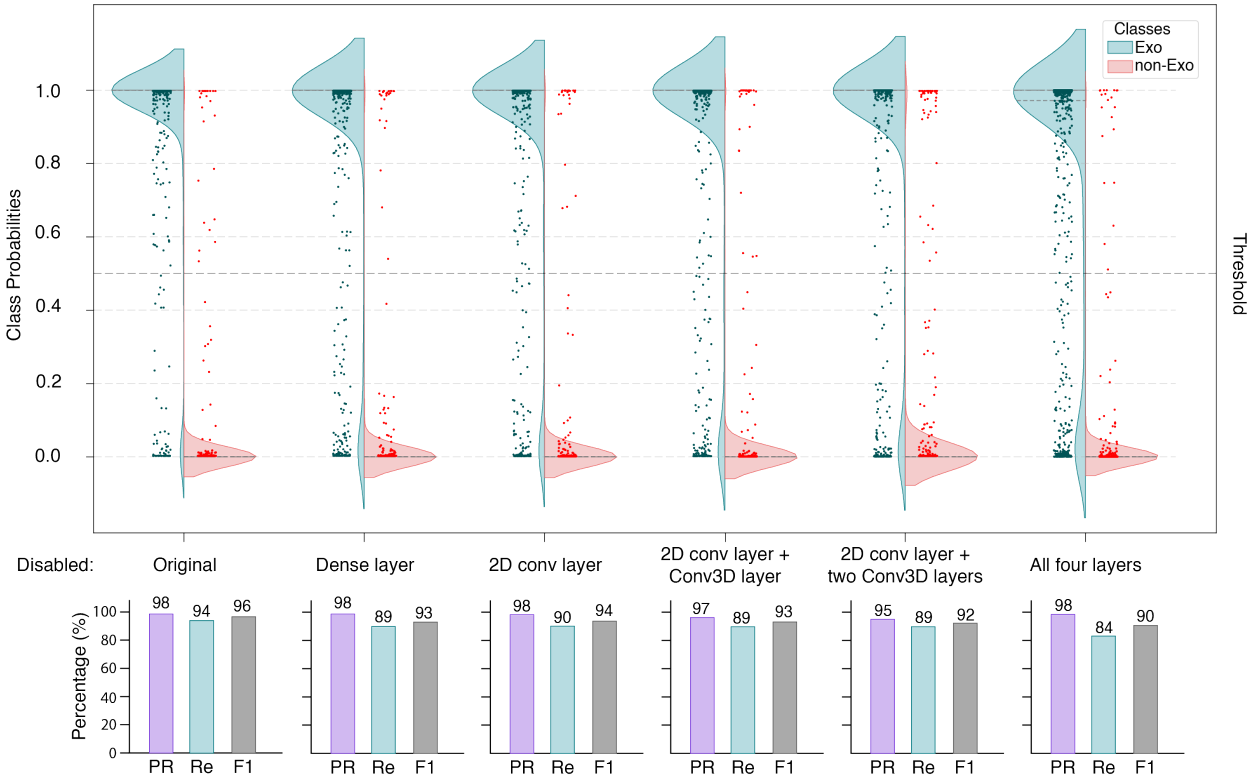

Supplementary Figure 15. Ablation study for the eViT model. This figure represents the results of ablation studies on an encoder vision transformer model, analyzing the impact of removing specific layers on classification performance. In this figure layers of the shared CNN and the second last Dense layer were progressively removed from the neural network before training. Each violin plot reflects the classes' probabilities derived from six independent training iterations (five retrained models plus the original model). Model class probabilities were evaluated across 2558 data points representing two classes (1467 non-exocytosis, red; 1091 exocytosis, turquoise). The accompanying bar graphs below each violin plot display the mean PR (purple), RE (blue), and F1 score (grey) values. The violin plots in both panels illustrate the variability in metric performance across the evaluated layers, while the bar graphs provide a clear comparative summary. Source data are provided as a Source Data file.

Supplementary Tables

Supplementary Table 1: IVEA available models with descriptions.

Analysis Module	Neural network	Model name	Description	Status
**Random**	eViT	GranuVision3	With cloud, without cloud, and latent granule fusion	Default
	eViT	GranuVision2	With cloud and without cloud	Option 2
	LSTM	GranuLSTM	Cloud, without cloud, and latent granule fusion	Option 3
**Stationary**	LSTM	NeuroLSTM	Burst events and slow events	Default

Supplementary Table 2: Human experts evaluating the movies. Initial evaluation was performed by the person who acquired the data and who was trained in recognizing exocytosis events.

Figure	Initial evaluation	IVEA validation
**3 a**	O. Khamis	A. Chouaib, U. Becherer
**3 b**	H.-F. Chang	A. Chouaib
**3 c**	N. Alawar, U. Becherer	A. Chouaib
**3 d**	S. Hugo[2], S. Echeverry, U. Becherer[1]	A. Chouaib, U. Becherer, S. Echeverry
**3 e**	S. Echeverry	A. Chouaib, S. Echeverry, S. Barg
**4 b, c**	A.S. Shaib[3], A. Staudt[4]	A. Chouaib, U. Becherer
**5 f**	S. Elizarova	A. Chouaib
**Supp. 4**	L. Demeersseman	A. Chouaib, L. Demeersseman
**Supp. 5**	U. Becherer[1]	A. Chouaib, U. Becherer
**Supp. 8**	Q. Tian	A. Chouaib, Q. Tian
**Supp. 10**	A. Staudt[4], U Becherer	A. Chouaib, U. Becherer

Supplementary Table 3 (related to Fig. 3): summary of the performance of our eViT, the LSM and ExoJ. In the "Quality" section, the presence of an increasing number of asterisks serves as an indication of enhanced performance (high recall, precision and F1 score) and, consequently, elevated functionality (batch analysis, automated parameters, ROI data availability). Conversely, the absence of asterisks signifies substandard performance, rendering the software unsuitable for the designated task.

	# of TP events	Recall (%)	Precision (%)	F1 score (%)	N_{cells}	Quality
**CTL expressing pH sensitive probe (Fig. 3a)**						
HE	77					
eViT	85	98.46 ± 1.48	97.48 ± 1.29	97.81 ± 0.98	13	*****
LSTM	70	86.81 ± 4.42	94.32 ± 3.78	89.41 ± 3.79	13	****
ExoJ	29	26.85 ± 5.73	66.64 ± 9.68	38.94 ± 6.40	13	*
**CTL expressing pH insensitive probe (Fig. 3b)**						
HE	168					
eViT	219	99.24 ± 0.34	81.71 ± 3.65	89.31± 2.12	33	*****
LSTM	172	89.14 ± 4.78	88.89 ± 2.32	88.48 ± 2.79	33	***
ExoJ	13	8.86 ± 2.67	11.14 ± 4.19	9.29 ± 2.76	33	
**CTL expressing CD63-SEP for long lasting events (Fig. 3c)**						
HE	86					
eViT	96	97.84 ± 1.39	98.58 ± 1.30	98.16 ± 1.07	6	*****
LSTM	78	90.79 ± 5.70	79.17 ± 10.90	83.26 ± 9.30	6	***
ExoJ	82	96.23 ± 2.71	87.48 ± 9.97	88.92 ± 7.28	6	***
**Chromaffin or INS1 cells expressing pH insensitive probe (Fig. 3d)**						
HE	292					
eViT	412	98.21 ± 6.64	78.27 ± 3.48	86.58 ± 9.81	10	****
LSTM	110	27.74 ± 6.64	93.49 ± 3.48	41.67 ± 2.79	10	**
ExoJ	4	1.2 ± 0.01	4.30 ± 3.34	10.00 ± 5.00	10	
**INS1 cells expressing probes with little pH sensitivity (Fig. 3e)**						
HE	147					
eViT	214	98.70 ± 0.93	90.08 ± 2.54	94.05 ± 1.53	8	*****
LSTM	66	29.89 ± 4.35	78.50 ± 8.67	40.08 ± 4.63	8	*
ExoJ	8	8.38 ± 3.88	25.25 ± 12.36	23.75 ± 7.00	8	

Supplementary Table 4 (related to Fig. 3): statistical comparison of our eViT with the LSM and ExoJ. Given are the p value of the performed tests. ANOVA on rank with Dunn's post-test is marked with an *, ANOVA with Holm-Sidak post-test is marked with # and Student two tailed paired t-test is marked with §. Non-significant differences are shown as ns. The p value is indicated in brackets when it is below 0.2. Pairs that could not be tested due to many missing values, are labeled with //.

	eViT to LSTM	eViT to ExoJ	LSTM to ExoJ	N
**CTL expressing pH sensitive probe (Fig. 3a)**				
Recall *	ns	<0.001	0.001	13
Precision *	ns	ns	ns (0.078)	13
F1 score *	ns	<0.001	0.001	13
**CTL expressing pH insensitive probe (Fig. 3b)**				
Recall *	ns	<0.001	0.020	7
Precision #	ns (0.155)	<0.001	<0.001	7
F1 score *	ns	<0.001	<0.001	7
**CTL expressing CD63 for long lasting events (Fig. 3c)**				
Recall *	ns	ns	ns	6
Precision #	ns	ns	ns	6
F1 score *	ns	ns	ns	6
**Chromaffin or INS1 cells expressing pH insensitive probe (Fig. 3d)**				
Recall *	0.033	<0.001	0.033	10
Precision *	ns (0.126)	0.016	<0.001	10
F1 score §	0.002	//	//	10
**INS1 cells expressing probes with little pH sensitivity (Fig. 3e)**				
Recall *	0.030	<0.001	ns	8
Precision #	ns	<0.001	0.002	8
F1 score §	0.00000793	//	//	8

Supplementary Table 5 (related to Fig. 3): Iterative adjustment of ExoJ analysis parameter. Ten different parameters can be adjusted in ExoJ to improve event detection. We found that the first 8 parameters (Min points for fitting procedure down to Max. displacement) did not alter event detection. In contrast the last two parameters, Min. R^2 (Decay fitting procedure, DFP)) and Min. R^2 (Est. radius fitting procedure, RFP), greatly affected event detection. We started the iterative adjustment with the default value then we first adjusted RFP and then DFP until the best possible detection was achieved. Metrics are: true positive (TP), false positive (FP) and false negative (FN) events. Test was performed on one movie from **Fig 3a**.

Metric	ExoJ							eViT
	Default 0.9 – 0.75	DFP – RFP 0.9 – **0.4**	DFP – RFP 0.9 – **0.5**	DFP – RFP 0.9 – **0.6**	DFP – RFP **0.8** – 0.5	DFP – RFP **0.9** – 0.5	DFP – RFP **0.95** – 0.5	Default
TP	3	5	5	3	5	5	2	14
FP	1	11	5	1	9	5	1	2
FN	11	9	9	11	9	9	12	0

Supplementary Table 6: Comparison of our eViT with pHusion. The results of both programs are shown for the movies of each CTL labeled with the pH sensitive GzmB-pHuji (**Fig. 3a**). Movies of cells that did not yield any result using pHusion are labeled with //. We adjusted the Grid spacing to 0.11 µm (pixel size) and in the "good trace" selection we used an R2 function value of 0.9. All other pHusion parameters were default parameters. Displayed are the result from "traces collapsed to single event". Shown are the true positive (TP), false positive (FP) and false negative (FN) events.

	pHusion software			IVEA (eViT)			HE
**Cells**	TP	FP	FN	TP	FP	FN	TP
**1**	//	//	//	4	0	0	4
**2**	//	//	//	4	0	0	4
**3**	//	//	//	4	0	0	3
**4**	//	//	//	2	0	0	2
**5**	0	0	16	16	0	0	14
**6**	//	//	//	10	1	0	7
**7**	1	3	3	4	0	0	2
**8**	14	2	0	14	2	0	14
**9**	6	3	2	8	1	0	8
**10**	4	3	3	7	0	0	7
**Total**	25	11	24	73	4	0	65

Supplementary Table 7: Number of events on which the eViT models and the LSTM models have been trained.

Neural network	Cell type	Indicator	Number of videos/events
**eViT**	CTL	pH sensitive (pHuji)	151/1156
	CTL	pH insensitive (tdTomato)	347/3806
	CTL or HEK cells	CD63-pHuji or SEP	47/309
	Chromaffin or INS cells	pH insensitive (mCherry)	50/2451
	Simulation videos	pH-sensitive (with cloud)	13/209
**LSTM**	DRG neuron	SypHy	39/11,300

Supplementary References

1. Becherer, U. et al. Quantifying exocytosis by combination of membrane capacitance measurements and total internal reflection fluorescence microscopy in chromaffin cells. *PLoS One* **2**, e505 (2007).
2. Hugo, S. et al. Deciphering dead-end docking of large dense core vesicles in bovine chromaffin cells. *J Neurosci* **33**, 17123-17137 (2013).
3. Shaib, A.H. et al. Paralogs of the Calcium-Dependent Activator Protein for Secretion Differentially Regulate Synaptic Transmission and Peptide Secretion in Sensory Neurons. *Front Cell Neurosci* **12**, 304 (2018).
4. Staudt, A. et al. Localization of the Priming Factors CAPS1 and CAPS2 in Mouse Sensory Neurons Is Determined by Their N-Termini. *Front Mol Neurosci* **15**, 674243 (2022).
