# Supplementary material for: Highly adaptable deep-learning platform for automated detection and analysis of vesicle exocytosis
Source: Nat Commun. 2025 Jul 12;16:6450. doi: 10.1038/s41467-025-61579-3 (PMC12255801; doi:10.1038/s41467-025-61579-3)
Supplement: Supplementary file 2 — Description of Additional Supplementary Files [file 41467_2025_61579_MOESM2_ESM.docx]

**Description of Additional Supplementary Files**

**File name: Chouaib et al - 2025 - Supplementary information**

Description: Contains the Supplementary Figures 1-15 and Supplementary Tables 1-7.

**File Name: Chouaib et al 2024 - Supplementary video 1.mp4**

Exemplary video of an exocytosis event of a lytic granule visualized through pH-sensitive fluorescent cargo protein.

**File Name: Chouaib et al 2024 - Supplementary video 2.mp4**

Description: Exemplary video of an exocytosis event of a lytic granule visualized with pH-insensitive fluorescent cargo protein.

**File Name: Chouaib et al 2024 - Supplementary video 3.mp4**

Description: Exemplary video of an exocytosis event of a lytic granule visualized with pH-insensitive fluorescent membrane protein.

**File Name: Chouaib et al 2024 - Supplementary video 4.mp4**

Description: Exemplary video of an exocytosis event of a lytic granule visualized with pH-sensitive fluorescent membrane protein.

**File Name: Chouaib et al 2024 - Supplementary video 5.mp4**

Description: Exemplary video of individual synaptic transmission events between dorsal root ganglion neurons and spinal cord neurons.

**File Name: Chouaib et al - 2025 - Source Data.xlsx**

Description: this is the Source data file
