# Supplementary material for: Highly adaptable deep-learning platform for automated detection and analysis of vesicle exocytosis
Source: Nat Commun. 2025 Jul 12;16:6450. doi: 10.1038/s41467-025-61579-3 (PMC12255801; doi:10.1038/s41467-025-61579-3)
Supplement: Supplementary file 8 — Reporting Summary [file 41467_2025_61579_MOESM8_ESM.pdf]

nature portfolio

Corresponding author(s): Ute Becherer and Ali Shaib

Last updated by author(s): Jun 9, 2025

Reporting Summary

Nature Portfolio wishes to improve the reproducibility of the work that we publish. This form provides structure for consistency and transparency in reporting. For further information on Nature Portfolio policies, see our Editorial Policies and the Editorial Policy Checklist.

Statistics

For all statistical analyses, confirm that the following items are present in the figure legend, table legend, main text, or Methods section.

n/a	Confirmed	
☐	☒	The exact sample size (*n*) for each experimental group/condition, given as a discrete number and unit of measurement
☐	☒	A statement on whether measurements were taken from distinct samples or whether the same sample was measured repeatedly
☐	☒	The statistical test(s) used AND whether they are one- or two-sided<br>*Only common tests should be described solely by name; describe more complex techniques in the Methods section.*
☐	☒	A description of all covariates tested
☐	☒	A description of any assumptions or corrections, such as tests of normality and adjustment for multiple comparisons
☐	☒	A full description of the statistical parameters including central tendency (e.g. means) or other basic estimates (e.g. regression coefficient) AND variation (e.g. standard deviation) or associated estimates of uncertainty (e.g. confidence intervals)
☐	☒	For null hypothesis testing, the test statistic (e.g. *F*, *t*, *r*) with confidence intervals, effect sizes, degrees of freedom and *P* value noted<br>*Give P values as exact values whenever suitable.*
☒	☐	For Bayesian analysis, information on the choice of priors and Markov chain Monte Carlo settings
☒	☐	For hierarchical and complex designs, identification of the appropriate level for tests and full reporting of outcomes
☒	☐	Estimates of effect sizes (e.g. Cohen's *d*, Pearson's *r*), indicating how they were calculated

Our web collection on statistics for biologists contains articles on many of the points above.

Software and code

Policy information about availability of computer code

Data collection	Microscopy data were collected using the proprietary software of the microscope manufacturer (Visiview V4.0.0.11 (Visitron-Systems GmbH), Methamorph (V7.8.0.0 and V7.10.5.476 Molecular Devices)).
Data analysis	IVEA was developed in Java 1.8.0_322 using Eclipse and the following Java libraries: ij (V1.54c), opencsv, bio-formats_plugins, loci_plugins, deeplearning4j core v1.0.0-M1.1, Google TensorFlow v1.15.0, libtensorflow_jni v1.15.0.<br>IVEA for training was done in Python v3.8.15 language using with the following libraries: Google TensorFlow v2.9.1 or v2.10, Keras, Numpy, Scikit-image, Tkinter, shutil, pandas, h5py, and read_roi.<br>IVEA training platform was coded with visual studio code V1.100.2<br>We used deep learning long-short term memory network, vision transformer network, convolution neural network, k-means clustering, iterative thresholding, Gaussian non-maximum suppression and multilayer intensity correction algorithm for software development.<br>Imaging data were analyzed by the human expert with Fiji V1.54p. The results were compared to ExoJ (V1.09), pHusion and SynActJ V0.3 software.<br>Data analysis was done in MATLAB (Mathworks 2024b) Excel 2021 (Microsoft). Statistic analysis was performed in SigmaPlot V14.5.0.101 (Systat Software, Inc.).

For manuscripts utilizing custom algorithms or software that are central to the research but not yet described in published literature, software must be made available to editors and reviewers. We strongly encourage code deposition in a community repository (e.g. GitHub). See the Nature Portfolio guidelines for submitting code & software for further information.

Data

Policy information about availability of data

All manuscripts must include a data availability statement. This statement should provide the following information, where applicable:

- Accession codes, unique identifiers, or web links for publicly available datasets
- A description of any restrictions on data availability
- For clinical datasets or third party data, please ensure that the statement adheres to our policy

Data availability: All original datasets used in this study, including the Source Data file, the labelled training data, and demonstration videos, have been deposited on Zenodo: https://doi.org/10.5281/zenodo.13153017. Data will be made available without restriction upon request via the Zenodo web interface. However, the underlying data for Figure 3 and Supplementary Data 4 will be provided without restriction only to programmers who require access to a large amount of data for testing and training new models. Source data are provided with this paper.

Code availability: The code used to develop the model, perform the analyses, and generate the results in this study is publicly available under the GPL v3.0 license at GitHub: https://github.com/AbedChouaib/IVEA. The version of the code associated with this manuscript has been archived on Zenodo at https://doi.org/10.5281/zenodo.15498139 (ref. 68).

Research involving human participants, their data, or biological material

Policy information about studies with human participants or human data. See also policy information about sex, gender (identity/presentation), and sexual orientation and race, ethnicity and racism.

Reporting on sex and gender	No individual-level data was provided in this study and thus sex/gender was not reported.
Reporting on race, ethnicity, or other socially relevant groupings	No individual-level data was provided in this study and thus race, ethnicity, or other socially relevant groupings was not reported.
Population characteristics	No individual-level data was provided in this study and population characteristics was not reported.
Recruitment	Peripheral blood samples from healthy donors were provided the Établissement Français du Sang (France) following the procedure agreed by the French Ministry of Education and Research. The donors of both blood centers were healthy adults of all ages, eligible to donated blood for transfusion and research purposes, and provided written consent for their blood samples to be used for research. Samples used in this manuscript were randomly selected from the daily pool of donors and sent to research labs as needed. As such, there was no discernible bias in sample selection and peripheral blood samples were fully de-identified prior to being received by research labs. No donor information and no medical records were obtained by the labs.
Ethics oversight	Deidentified blood samples were collected and processed following standard ethical procedures after obtaining written informed consent from each donor and approval by the French Ministry of the Research as described (Cortacero et al. 2023, authorization no. DC-2021-4673).

Note that full information on the approval of the study protocol must also be provided in the manuscript.

Field-specific reporting

Please select the one below that is the best fit for your research. If you are not sure, read the appropriate sections before making your selection.

[x] Life sciences [ ] Behavioural & social sciences [ ] Ecological, evolutionary & environmental sciences

For a reference copy of the document with all sections, see nature.com/documents/nr-reporting-summary-flat.pdf

Life sciences study design

All studies must disclose on these points even when the disclosure is negative.

Sample size	The program was tested on at least 10 cells per cell type and labeling condition. The eViT was tested on 8 cell type and labeling conditions. The LSTM was tested on 9 cell types. Training of the eViT was performed on 548 videos with approximately 7k data samples. The LSTM was trained on 11.3k data samples.<br>Statistical tests (ANOVA, ANOVA on ranks and Student's t-test) were applied according to the data, the outcome and the test are given in the supplementary table 1
Data exclusions	Movies that showed 0 or 1 exocytotic events or with very low sampling rate (below 2Hz) or with large experimental artifacts such as focus change or drift, were exclude so as to generate meaningful results.
Replication	Not applicable because this is a computational study
Randomization	The allocation of images for training and testing the neural networks was random
Blinding	Not applicable because this is a computational study

Reporting for specific materials, systems and methods

We require information from authors about some types of materials, experimental systems and methods used in many studies. Here, indicate whether each material, system or method listed is relevant to your study. If you are not sure if a list item applies to your research, read the appropriate section before selecting a response.

Materials & experimental systems

n/a	Involved in the study
☒	☐ Antibodies
☐	☒ Eukaryotic cell lines
☒	☐ Palaeontology and archaeology
☐	☒ Animals and other organisms
☒	☐ Clinical data
☒	☐ Dual use research of concern
☒	☐ Plants

Methods

n/a	Involved in the study
☒	☐ ChIP-seq
☒	☐ Flow cytometry
☒	☐ MRI-based neuroimaging

Eukaryotic cell lines

Policy information about cell lines and Sex and Gender in Research

Cell line source(s)	INS-1 cells (clone 832/13)
Authentication	The cell line was provided by Hendrik Mulder (Lund University).
Mycoplasma contamination	The cells are regularly tested for mycoplasma using PCR.
Commonly misidentified lines (See ICLAC register)	Not listed in the ICLAC database.

Animals and other research organisms

Policy information about studies involving animals; ARRIVE guidelines recommended for reporting animal research, and Sex and Gender in Research

Laboratory animals	WT mice with C57BL/6N background used in this study were purchased from Charles River while Granzyme B-tdTomato KI mice40 were purchased from the Transgenesis and Archiving of Animal Models (TAAM) (National Centre of Scientific Research (CNRS), Orleans, France). Mice were of either sex were used at the age of 0-22 weeks depending on the required cell.
Wild animals	None
Reporting on sex	Both sexes
Field-collected samples	None
Ethics oversight	Overall the study did not require specific ethic oversight. All experimental procedures were approved and performed according to the regulations by the state of Saarland (Landesamt für Verbraucherschutz, AZ.: 2.4.1.1). Ethical guidelines for the care and use of laboratory animals, issued by the German Government and approved by the Commissions for Institutional Animal Care and Use at Saarland University, Saarland, Germany, were followed (animal license number 41–2016 and 52/2012) .

Note that full information on the approval of the study protocol must also be provided in the manuscript.

Plants

Seed stocks	Not relevant for this study.
Novel plant genotypes	Not relevant for this study.
Authentication	Not relevant for this study.
